# A tris-spiro metalla-aromatic system featuring Craig-Möbius aromaticity

**DOI:** 10.1038/s41467-021-21648-9

**Published:** 2021-02-26

**Authors:** Zhe Huang, Yongliang Zhang, Wen-Xiong Zhang, Junnian Wei, Shengfa Ye, Zhenfeng Xi

**Affiliations:** 1 Beijing National Laboratory for Molecular Sciences (BNLMS), Key Laboratory of Bioorganic Chemistry and Molecular Engineering of Ministry of Education, College of Chemistry, Peking University, Beijing, 100871 China; 2 State Key Laboratory of Catalysis, Dalian Institute of Chemical Physics, Chinese Academy of Sciences, 457 Zhongshan Road, Dalian, 116023 China; 3 Max-Planck-Institut für Kohlenforschung, Kaiser-Wilhelm-Platz 1, D-45470, Mülheim an der Ruhr, Germany; 4 State Key Laboratory of Organometallic Chemistry, Shanghai Institute of Organic Chemistry, Chinese Academy of Sciences, Shanghai, 200032 China

**Keywords:** Coordination chemistry, Organometallic chemistry, Density functional theory

## Abstract

As aromaticity is one of the most fundamental concepts in chemistry, the construction of aromatic systems has long been an important subject. Herein, we report the synthesis and characterization of a tris-spiroaromatic complex, hexalithio spiro vanadacycle **2**. The delocalization of the four electrons within the two V 3*d* orbitals and the π* orbitals of the three biphenyl ligands leads to a 40π Craig-Möbius aromatic system with three metalla-aromatic rings, as revealed by both experimental measurements and theoretical analyses. For comparison, if Cr is used instead of V, a similar Craig-Möbius aromatic system can not be generated. In this case, pentalithio spiro chromacycle **3** is obtained, and the Cr center uses its two 3*d* orbitals to form two independent metalla-aromatic rings. This work presents a type of aromatic systems that will contribute to both aromaticity theory and organometallic chemistry.

## Abstract (web summary)

Spiroaromatic compounds are advantageous platforms for designing expanded aromatic systems. Herein, the authors present a tris‐spiro metalla‐aromatic Vanadium compound which forms a 40π Craig‐Möbius aromatic system.

## Introduction

**Fig. 1:**
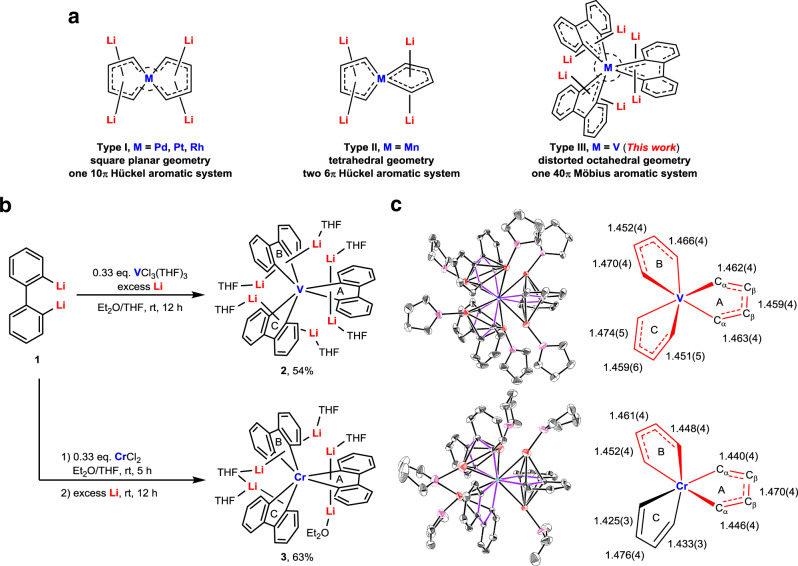
Synthesis and molecular structures of hexalithio spiro vanadacycle 2 and pentalithio spiro chromacycle 3. **a**, Three types of spiro metalla-aromatics. **b**, Synthesis of complexes **2** and **3**. **c**, X-ray molecular structures of complexes **2** and **3** drawn with 30% thermal ellipsoids and selected bond lengths (Å). Hydrogen atoms are omitted for clarity. Metalla-aromatic rings are drawn in red.

Aromaticity is one of the most long-standing and fundamental concepts in chemistry^1–7^. While the original studies mainly focused on conventional organic aromatic compounds, the introduction of transition metals into aromatic systems has brought about fascinating metalla-aromatic structures that are distinct from their corresponding hydrocarbon analogs, mostly because of the participation of *d* orbitals^8–19^. For example, the term spiroaromaticity was first introduced to describe spirocyclic compounds with homoconjugation, in which the spiro *sp*^3^ carbon atom is not involved^20–22^. In 2002, Rzepa proposed that the spiro atom itself could participate in the conjugation to form a class of spiroaromatic systems in which each ring could sustain aromaticity independently^23,24^ or join together to exhibit global aromaticity^25^. This type of spiroaromaticity could not be achieved when the spiro atom is carbon, which has only four valence orbitals. However, by employing transition metals as the spiro atom, we have realized two types of bis-spiro metalla-aromatics with square planar (Type I, Fig. 1a)^18^ and tetrahedral (Type II, Fig. 1a) geometries^19^. The *d* electron configurations of the metal centers were found to be a critical factor that largely dictates their geometric and electronic structures. Accordingly, we surmised that to further construct tris-spiro metalla-aromatic structures, the metal center needs to possess more empty *d*-orbitals, which prompted us to turn our attention to early transition metals.

Rzepa et al. have theoretically studied a number of tris-spiroaromatic candidates having P, As, or V as the spiro atom, which exhibit a certain degree of aromaticity^24,25^, but none of them has been realized yet. Although Craig-Möbius type molecular orbitals were found in Ta(DAD)_3_^+^ (DAD = 1,4-diazabuta-1,3-diene)^25,26^, no other proofs have been provided to support its aromaticity. Besides, the presence of Möbius type orbitals in metallacycles is only a necessary condition, rather than a sufficient one, for Möbius aromaticity, because Hückel aromatic systems with transition metals may also involve such orbitals, as demonstrated by Mauksch and Tsogoeva^9^.

Herein we report hexalithio spiro vanadacycle **2** as a tris-spiro metalla-aromatic compound (Type III, Fig. 1a). The V atom uses its two 3*d* orbitals to interact with the π* orbital of the three biphenyl ligands; consequently, the resulting three metalla-aromatic rings as a whole lead to a 40π Craig-Möbius aromatic system. For comparison, pentalithio spiro chromacycle **3** is also synthesized, in which the Cr atom employs its two 3*d* orbitals to form two independent metalla-aromatic rings.

## Results

### Synthesis

Although the previous spiro metalla-aromatics were synthesized from 1,4-dilithio-1,3-butadienes in our group^18,19^, similar attempts with early transition metals failed, probably due to the presence of redox side-reactions. We then turned to 2,2′-dilithiobiphenyl **1**^27,28^, another potential non-innocent ligand with a similar π* orbital, which has been utilized to synthesize aromatic 9-germafluorenyl^29^ and 9-stannafluorenyl dianions^30^. As shown in Fig. 1b, the reaction of **1** with 0.33 equivalents of VCl_3_(THF)_3_ and an excess amount of metallic lithium in the mixed solvent of Et_2_O and THF (3:1) at room temperature afforded hexalithio spiro vanadacycle **2** in 54% isolated yield as dark blue crystals. Although a similar three-component reaction using CrCl_2_ instead of VCl_3_(THF)_3_ did not give any isolable product, the stepwise reaction of **1** with CrCl_2_ and lithium could provide pentalithio spiro chromacycle **3** as dark green crystals in 63% yield.

### Characterization

The single crystals of **2** and **3** could be obtained by recrystallization in the mixed solvent of hexane, Et_2_O, and THF at −20 °C. Their molecular structures were determined by X-ray single-crystal structural analyses. As shown in Fig. 1c, complex **2** features three vanadacycles spiro-fused by one V atom. The averaged C–V–C angle within the vanadacycles is 74.7°, and the averaged *trans* C–V–C angle is 160.9°, indicating a distorted octahedral geometry with approximate *D*_3_ symmetry. For each vanadacycle there are two Li cations bonded in *η*^5^ mode. All three vanadacycles are nearly planar with almost vanishing folding angles (0.1°, 1.7°, and 0.5°). The C–C bond lengths within the 5-membered rings are remarkably averaged compared to those in **1** (1.4305(19), 1.513(2), 1.4295(18) Å). Meanwhile, the C–C bond length alternation in the phenyl rings increases slightly (Supplementary Fig. 3). These features indicate considerable electron back donation from the V center to the biphenyl ligands and thus hint at a possible metalla-aromatic structure^29,30^. Organovanadium complexes with six V–C σ bonds are very rare^31^, complex **2** is a structurally well-defined tris-spiro vanadacycle with six V–C σ bonds.

As shown in Fig. 1c, complex **3** also has a tris-spiro scaffold. Two of the chromacycles (Rings A and B) have two *η*^5^ bonded Li cations, while the other one (Ring C) has only one *η*^5^ bonded Li cation, which is coordinated by two THF molecules. This might be resulted from the relatively weaker interaction between the Li cation and “monoanionic” Ring C, as reflected by the larger Li–ring distance (2.082 Å) compared to those of Ring A (avg. 1.874 Å) and Ring B (avg. 1.848 Å). One of the Li cations bonded to Ring A is coordinated by Et_2_O, probably due to the reduced steric hindrance. Rings A and B have nearly planar structures with averaged C–C bonds within the 5-membered rings, which is similar to the vanadacycles in **2** but distinct from the reported “neutral” chromafluorene (1.419(4), 1.464(4), 1.425(4) Å)^32^. However, Ring C has a larger folding angle (8.4°) and a C–C bond alternation analogous to that reported for non-aromatic dilithionickelafluorene (1.438(5), 1.479(6), 1.427(5) Å)^17^. These features imply that Rings A and B have a metalla-aromatic structure whereas Ring C does not.

**Fig. 2:**
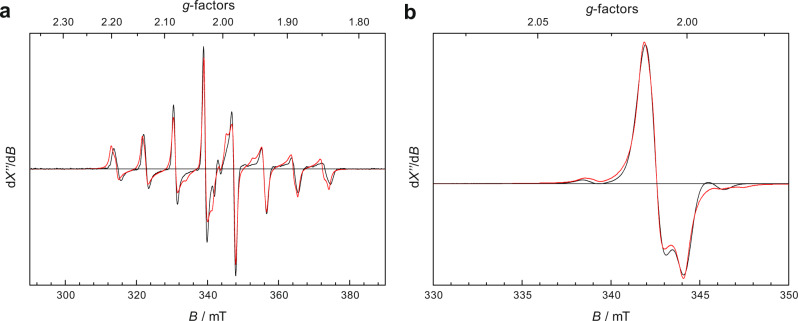
X-band EPR spectra of complexes 2 and 3 (black trace) and simulation (red trace). **a** Spectrum of **2** recorded at 30 K. **b** Spectrum of **3** recorded at 40 K.

As both **2** and **3** have an odd number of electrons, EPR spectroscopy was employed to probe their electronic structures. The measured EPR spectrum of **2** (Fig. 2a) at the base temperature showed an octet signal centered at *g* ~ 2.0, thereby indicating that **2** possesses an *S* = 1/2 ground state as [V(bpy)_3_]^33^ and [V(^*t*^bpy)_3_]^34^ (bpy = 2,2′-bipyridine, and ^*t*^bpy = 4,4′-di-*tert*-butyl-2,2′-bipyridine), which elicit similar EPR spectra. The eight lines are due to the hyperfine splitting of ^51^V (*I* = 7/2, 99.75% abundance). Simulations yielded *g*_x,y,z_ = 2.006, 2.005, 2.005, and A_x,y,z_ = 77.2, 81.8, 33.1 × 10^−4^ cm^−1^. The observed large magnitude of *A* suggested that the unpaired electron primarily resides in a V 3*d* based orbital. In analogy to [Cr(^*t*^bpy)_3_](PF_6_)^35^, the EPR spectrum of **3** (Fig. 2b) exhibited a nearly axial feature at *g* ~ 2.0 flanked by a pair of satellite peaks at both sides originating from the hyperfine interaction of ^53^Cr (*I* = 3/2, 9.50% abundance). Consequently, **3** also features a doublet ground state. Simulations gave *g*_x,y,z_ = 2.015, 2.010, 2.001, and *A*_iso_ = 21.3 × 10^−4^ cm^−1^. The large hyperfine coupling constant of ^53^Cr demonstrates that the unpaired electron in **3** is predominantly located in the Cr center as found for [Cr(^*t*^bpy)_3_](PF_6_)^35^.

To gain more insight into the oxidation states of the metal centers in **2** and **3**, X-ray photoelectron spectroscopy (XPS) experiments were also carried out. The XPS of **2** detected the V 2*p*_3/2_ binding energy (BE) at 517.2 eV (Supplementary Fig. 7). Although no octahedral V complex with similar BE was found in the NIST XPS database, this BE value is notably higher than those of K_4_[V(CN)_6_] (513.3 eV)^36^ and V(acac)_3_ (514.2 eV)^37^ as archetypal octahedral V(II) and V(III) complexes, which suggests that the oxidation state of V in **2** is most likely higher than +3. The Cr 2*p*_3/2_ BE of **3** was observed at 577.5 eV (Supplementary Fig. 8), similar to those of Cr(SCN)_6_(PPh_4_)_3_ (577.4 eV)^38^ and Cr(acac)_3_ (577.7 eV)^39^, thereby indicating a Cr(III) center in **3**.

### DFT calculations

**Fig. 3:**
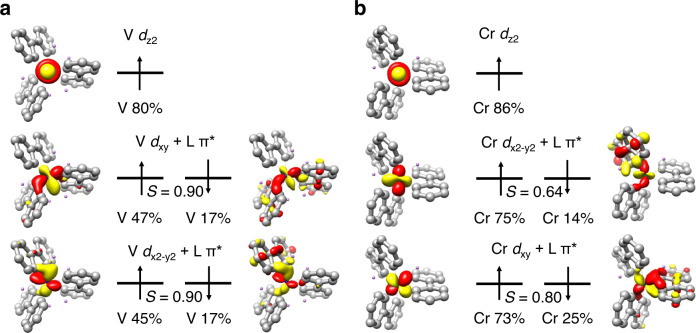
Schematic MO diagrams. *S* is the spatial overlap between the two orbitals of a given spin coupled pair. Schematic MO diagrams of complexes 2 (**a**) and 3 (**b**).

Based on the experimental findings, DFT calculation was carried out to further elucidate the electronic structures of **2** and **3**. The optimized structures of **2** and **3** agree well with their single crystal structures (Supplementary Tables 4 and 5). As shown in Fig. 3a, in the upper valence region of complex **2**, one can identify five singly occupied molecular orbitals (MOs) with considerable V 3*d* character. Among them, one is an essential nonbonding V *d*_z2_ based orbital with predominant V parentage (80%). The other four MOs form two spin coupled pairs and represent the bonding combination between the V *d*_xy_ and *d*_x2-y2_ orbitals and the group orbital of e symmetry composed of the π* orbitals of the three biphenyl ligands^40^. It should be noted that the V contributions in these four MOs are less than 50%. In other words, the four electrons are delocalized on the V center and the three ligands. This result indicates that the metal-ligand interaction is rather covalent; thus, the physical oxidation state of the V center cannot be unambiguously assigned, and the bonding of **2** is best interpreted as a resonance hybrid of a range of limiting electronic-structure descriptions (Supplementary Fig. 12)^41^. Despite this, on the grounds of the computed V percentage, the one involving a 3*d*^1^ V(IV) center makes a dominant contribution to the resonance hybrid, consistent with the EPR and XPS measurements. Specifically, the observation that the unpaired electron of **2** predominantly locates in the *d*_z2_ centered singly occupied MO is consistent with its *A*_x,y,z_ matrix with two large and one small components. Because the biphenyl π* orbital is bonding in nature with respect to the C_β_–C_β_ bond but antibonding with respect to the C_α_–C_β_ bond, the electron population of this π* orbital should lead to a lengthening of the C_α_–C_β_ bond and shortening of the C_β_–C_β_ bond, which accounts for the observed equalization of the C–C bond lengths of the three vanadacycles in **2**.

As shown in Fig. 3b, complex **3** also has five singly occupied MOs with considerable Cr 3*d* character, and one of them is the nonbonding Cr *d*_z2_ based orbital. The other four MOs have negligible contribution from Ring C since the Cr *d*_xy_ and *d*_x2-y2_ orbitals preferentially interact with the π* orbitals of Rings A and B, a situation different from **2**. This can be traced back to the more pronounced geometric distortions of **3** from the idealized *D*_3_ symmetry. In these four MOs, the two spin-up ones possess dominant Cr percentages (> 70%), while the two spin-down ones are essentially the biphenyl π* orbitals. The electronic structure of **3** is thus best viewed as having a high-spin Cr(III) center that is antiferromagnetically coupled to two trianionic biphenyl radicals and chelated by another closed-shell dianionic ligand yielding an overall doublet ground state, viz. Li_5_Cr(III)(L^2-^)(L^•3−^)_2_. This bonding description agrees with that deduced from the EPR and XPS measurements, and is reminiscent of our previous reported spiroaromatic manganacycle with two independent 6π aromatic rings^19^.

According to our previous investigations^17–19^, strong *d*-π* interactions between the metal center and the ligands as those found for **2** and **3** might induce metalla-aromaticity. To further verify this hypothesis, a series of theoretical methods were employed. The NICS value, a widely used index to evaluate aromaticity^42,43^, was calculated for the five-membered metallacycles in **2** and **3**. The calculated NICS(1)_zz_ values for the three vanadacycles in **2** were −24.9, −26.0, and −24.4 ppm, indicating three metalla-aromatic rings. For complex **3**, Rings A and B are metalla-aromatic as suggested by the estimated NICS(1)_zz_ values of −18.5 and −19.5 ppm, while Ring C with an almost vanishing NICS(1)_zz_ value of −3.1 ppm is essentially non-aromatic. A similar trend was found for the calculated NICS(0) and NICS(0)_zz_ values (Supplementary Table 6). For comparison, we also conducted a computational study on the aforementioned Ta(DAD)_3_^+^, which was proposed to be a tris-spiroaromatic species by Rzepa^25^. However, the NICS(1)_zz_ values turned out to be −1.2 ppm for all its three metallacycles, indicating non-aromaticity. This difference might arise from the lower π-HOMO-LUMO gap of dilithiobiphenyl **1** (4.42 eV) compared with DAD ligand (5.87 eV), which leads to an increasing covalency of the metal-ligand interaction and a higher degree of electron delocalization in complex **2**.

**Fig. 4:**
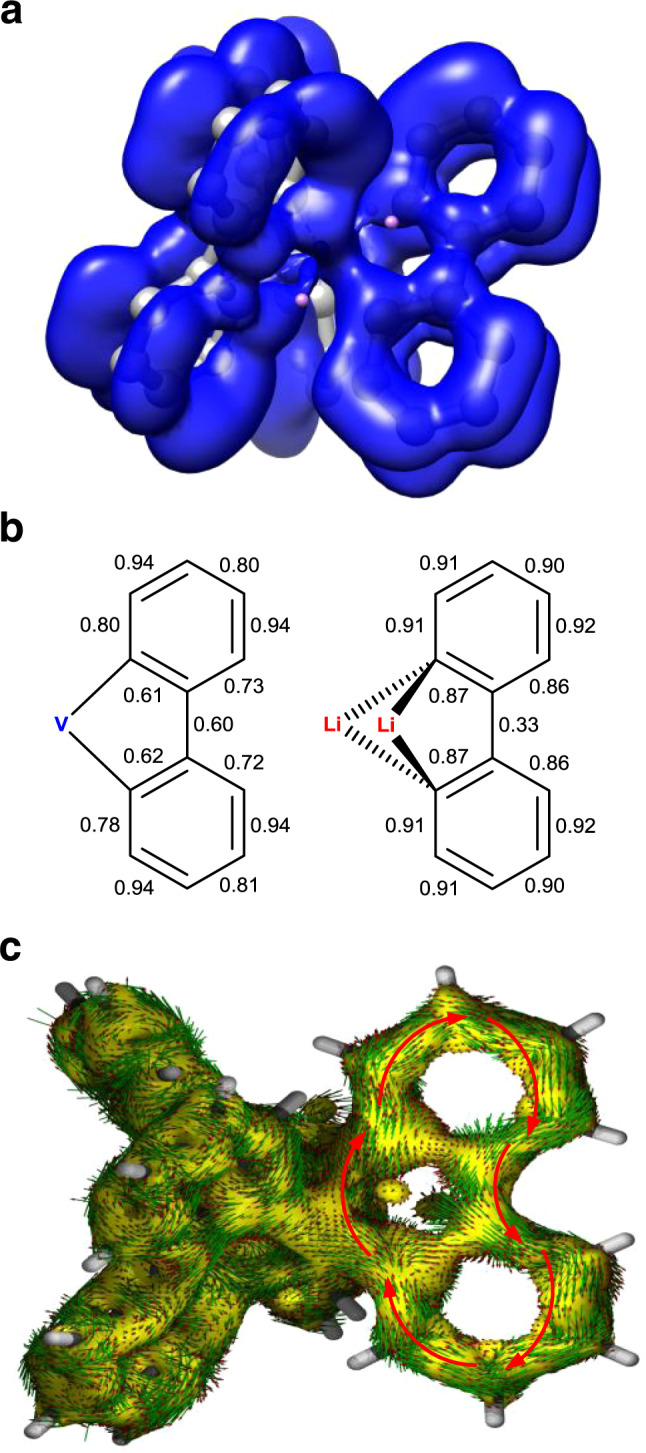
Analyses on the aromaticity of complex 2. **a** The ELF-π isosurface of **2** at 0.35. **b** ELF-π bifurcation values of the biphenyl ligands in **2** (Ring A) and **1**. **c** AICD plot of **2** from π contribution. AICD isosurface of 0.03 is shown in yellow. Red arrows represent the direction of the ring current. The magnetic field vector is orthogonal to the ring plane and points outside (clockwise currents are diatropic).

For complex **2**, in addition to the four electrons in the *d*-π* bonding orbitals shown in Fig. 3a, each biphenyl ligand also possesses 12 π electrons, thereby leading to a 40π Craig-Möbius aromatic system. Correspondingly, 20 pairs of singly occupied π-type MOs could be identified for **2** (20 spin-up and 20 spin-down, Supplementary Fig. 13), based on which the ELF-π analysis^44,45^ was undertaken. As shown in Fig. 4a, there is a substantial electron delocalization in the π orbitals of the three ligands circulated by the V *d* orbitals. The bifurcation values (BVs) of ELF-π on the three ligands are similar (Supplementary Fig. 14), suggesting an almost equal electron delocalization on them, and the BVs on Ring A is shown in Fig. 4b as an example. For comparison, the ELF-π BVs of dilithiobiphenyl **1** were also calculated and the result revealed that the electron delocalization on two phenyl rings is independent. Compared with **1**, complex **2** has smaller BVs at C_α_–C_β_ (**1**: 0.87; **2**: 0.61, 0.62) and a larger BV at C_β_–C_β_ (**1**: 0.33; **2**: 0.60), consistent with the single crystal bond lengths and the considerable electron density in the π* orbitals. This difference demonstrates that the strong *d*-π* interaction in **2** results in complete π electron delocalization over the entire complex and thus a metalla-aromatic structure.

AICD plot is a widely applied method for visualizing induced ring current^46,47^. Based on the 20 pairs of π-type MOs, AICD-π analysis was performed. As shown in Fig. 4c, when an external magnetic field is applied perpendicular to the vandacycle (e.g., Ring A in **2**), a strong diamagnetic ring current is generated over the V atom and the ligand, indicating a metalla-aromatic system.

As the coordinated Li cations in the dilithiometallole have been found to be indispensable to metalla-aromaticity in our previous work^19^, we also examined the hypothetical hexaanion **2′** by removing the six Li cations and THF in **2** without further geometry optimization. As a result, only one *d*-π* bonding orbital involving Rings A and C was found in **2′** (Supplementary Fig. 16). The calculated NICS(1)_zz_ values indicated that Ring A (−12.7, 4.0 ppm) and Ring C (−13.5, 0.3 ppm) in **2′** have negligible aromatic character while Ring B (−2.5, 1.1 ppm) is essentially non-aromatic (Supplementary Fig. 17). In addition, the geometry optimization starting from **2′** was also conducted, and the resulting species **2″** features remarkable C–C bond alternation in Rings A and B (Supplementary Fig. 18). These results reveal that the Li cations increase the electron affinity of the three dianoinic biphenyl ligands and strengthen the electron back donation from the V center to them. Consequently, the enhanced electron delocalization between the metal center and the ligands leads to 40π Craig-Möbius aromaticity of **2**.

Complexes **2** and **3** have a similar scaffold, but differ by the number of coordinated Li cations, we thus surmise that the latter could be a key factor to dictate their distinct aromaticity. Indeed, when adding one Li cation to Ring C in **3**, the resulting hypothetical cation **4** features three metalla-aromatic rings in analogy to **2**, as indicated by the calculated NICS values (Supplementary Table 6) and the averaged C–C bond lengths (Supplementary Table 7). Although **4** and **2** feature an analogous bonding situation, the four *d*-π* MOs in **4** possess more Cr *d* character (72%, 72%, 24%, 24%, Supplementary Fig. 19), suggesting weaker Cr-to-biphenyl ligand back donation in **4**. This largely arises from the higher effective nuclear charge of Cr than that of V. Consequently, the lower energy of the Cr orbitals diminishes the extent of the back donation. This results in a lower degree of the electron delocalization in chromacycles, consistent with their less negative NICS values compared to those found for vanadacycles. In addition, we also examined the hypothetical anion **5** by removing one Li cation from Ring C in **2** and a similar trend was observed between **3** and **5** (Supplementary Table 6, Supplementary Fig. 19). These findings further corroborate the notion that the presence of Li cations is crucial for the extended aromaticity, and underscore the important role played by spiro atoms, which can influence the degree of the electron delocalization.

In summary, hexalithio spiro vanadacycle **2** was synthesized and characterized to be a tris-spiroaromatic complex. The V center uses its two 3*d* orbitals to interact with π* orbitals of the three biphenyl ligands, forming a 40π Craig-Möbius aromatic system. Pentalithio spiro chromacycle **3** was also prepared, in which preferential interaction of the two Cr 3*d* orbitals with two biphenyl ligands leads to the formation of two independent metalla-aromatic rings. This work presents a class of spiroaromatic complexes and highlights the pivotal role of transition metals, due to its flexible bonding modes, in realizing aromaticity.

## Methods

### General information

Unless otherwise noted, all starting materials were commercially available and were used without further purification. Solvents were purified with an Mbraun SPS-800 Solvent Purification System. All reactions were carried out under a dry and oxygen-free nitrogen atmosphere by using Schlenk techniques or under an argon atmosphere in a Vigor (SG1200/750TS-F) glovebox. The argon in the glovebox was constantly circulated through a copper/molecular sieve catalyst unit. The oxygen and moisture concentrations in the glovebox atmosphere were monitored by an O_2_/H_2_O Combi-Analyzer to ensure both were always below 1 ppm. Elemental analysis was carried out using an Elementar Vario EL Cube elemental analyser. XPS was carried out on an Axis Ultra imaging photoelectron spectrometer. Solution magnetic susceptibility measurements were performed by the Evans method^48^ using a Bruker ARX400 spectrometer. IR spectra were recorded using a Bruker ALPHA II FTIR spectrometer. UV–vis spectra were recorded using an Agilent Cary 60 UV–vis spectrophotometer. X-band cw-EPR measurements were performed on a Bruker E500 ELEXSYS spectrometer equipped with the Bruker dual-mode cavity (ER4116DM) and an Oxford Instruments helium flow cryostat (ESR 900). The microwave bridge was the high-sensitivity bridge Super-X from Bruker (ER-049X) with an integrated microwave frequency counter. The magnetic field controller (ER032T) was externally calibrated with a Bruker NMR field probe (ER035M). The spin Hamiltonian simulations were performed with the program esimX developed by Dr. E. Bill.

### Synthesis of complex 1^49^

Under a nitrogen atmosphere, ^*n*^BuLi in hexane (7.8 mL, 2.7 mol/L, 21 mmol) was added dropwise to a solution of 2,2′-dibromo-1,1′-biphenyl (3.120 g, 10 mmol) in diethyl ether (50 mL) at −78 °C. The mixture was stirred at −78 °C for 30 min and then warmed to room temperature and stirred for 3 h. The solvent was removed in vacuum and the residue was washed with hexane (15 mL × 3) and dried under vacuum. Complex **1** was obtained as a white solid. Yield: 1.560 g, 94%.

### Synthesis of complex 2

In an argon atmosphere glovebox, a suspension of dilithiobiphenyl **1** (0.0747 g, 0.45 mmol) in Et_2_O (4 mL) was added into the mixture of VCl_3_(THF)_3_ (0.0560 g, 0.15 mmol) and metallic lithium (0.0345 g, 5.0 mmol) in Et_2_O (2 mL). Then THF (2 mL) was added drop by drop and the mixture was stirred at room temperature for 12 h. The solvent was removed under vacuum and the residue was extracted with toluene and filtered. The filtrate was recrystallized in the mixed solvent of hexane/Et_2_O/THF (10:10:1) at −20 °C and complex **2** was obtained as dark blue crystals. The crystals were washed with cold hexane and dried under vacuum. Yield: 79.9 mg, 54%. Analysis (calcd., found for C_60_H_72_Li_6_O_6_V): C (73.40, 73.24), H (7.39, 7.55). Magnetic susceptibility (Evans’method): *μ*_eff_ = 2.1 ± 0.1 *μ*_B_ in THF-d_8_ at 298 K. IR (KBr, cm^−1^) υ: 3020 (m), 2975 (m), 2926 (m), 2876 (m), 1547 (w), 1487 (w), 1388 (s), 1269 (s), 1228 (m), 1195 (m), 1140 (m), 1041 (m), 954 (s), 936 (s), 900 (s), 725 (s), 471 (w).

### Synthesis of complex 3

In an argon atmosphere glovebox, a suspension of dilithiobiphenyl **1** (0.0747 g, 0.45 mmol) in Et_2_O (4 mL) was added into the mixture of CrCl_2_ (0.0184 g, 0.15 mmol) and Et_2_O (2 mL). Then THF (2 mL) was added drop by drop. After stirred at room temperature for 5 h, metallic lithium (0.0345 g, 5.0 mmol) was added. and the mixture was stirred at room temperature for 12 h. The solvent was removed in vacuum and the residue was extracted with toluene and filtered. The filtrate was recrystallized in the mixed solvent of hexane/Et_2_O/THF (10:10:1) and complex **3** was obtained as dark green crystals. The crystals were washed with cold hexane and dried under vacuum. Yield: 85.9 mg, 63%. Analysis (calcd., found for C_56_H_66_CrLi_5_O_5_): C (74.25, 73.63), H (7.34, 7.31). A satisfying result for carbon content of **3** could not be obtained due to its extreme sensitivity to air. Magnetic susceptibility (Evans’method): *μ*_eff_ = 3.0 ± 0.1 *μ*_B_ in THF-d_8_ at 298 K. IR (KBr, cm^−1^) υ: 3026 (m), 2975 (m), 2925 (s), 2877 (m), 2854 (m), 1550 (m), 1459 (m), 1410 (m), 1390 (s), 1271 (s), 1228 (m), 1203 (m), 1198 (m), 1146 (m), 1042 (s), 957 (s), 909 (m), 891 (m), 738 (s), 729 (s), 466 (w), 454 (w).

### Computational details

DFT calculations were carried out using the ORCA quantum chemical program package^50^. Geometry optimizations were performed with the (U)B3LYP^51,52^ density functional in combination with D3(BJ) dispersion correction^53,54^. Vanadium, chromium, and tantalum atoms were treated with a Stuttgart effective core potential^55,56^ and Pople’s basis set 6-311 G(d,p)^57^ was used for other elements. ELF-π analysis was performed using the Multiwfn program^58^.

## Supplementary information

Supplementary Information

Description of Additional Supplementary Files

Supplementary Data 1

## Data Availability

Crystallographic data for the structures reported in this article have been deposited at the Cambridge Crystallographic Data Centre under deposition numbers CCDC 2033720 (**1**), CCDC 1984387 (**2**), and CCDC 1984388 (**3**) and are available from CCDC in cif format. These data can be obtained free of charge from The Cambridge Crystallographic Data Centre via www.ccdc.cam.ac.uk/data_request/cif. All other data supporting the findings of this study are available within the article and its Supplementary Information, or from the corresponding author upon reasonable request. Cartesian coordinates of the DFT optimized structures are provided in Supplementary Data 1.
