# Supplementary material for: A tris-spiro metalla-aromatic system featuring Craig-Möbius aromaticity
Source: Nat Commun. 2021 Feb 26;12:1319. doi: 10.1038/s41467-021-21648-9 (PMC7910433; doi:10.1038/s41467-021-21648-9)
Supplement: Supplementary file 1 — Supplementary Information [file 41467_2021_21648_MOESM1_ESM.pdf]

Supplementary Information

A tris-spiro metalla-aromatic system featuring Craig-Möbius aromaticity

Zhe Huang, Yongliang Zhang, Wen-Xiong Zhang, Junnian Wei*, Shengfa Ye* & Zhenfeng Xi*

Supplementary Methods

Single-Crystal X-Ray Diffraction Experiments

Although the crystal structure of TMEDA-coordinated 2,2'-dilithiobiphenyl (TMEDA= N,N,N',N'-tetramethylethylenediamine) has been reported in 1982[1], its limited structure resolution only allows for determination of the atom connectivity. Thereby we managed to obatin a high-quality crystal structure of THF-solvated 2,2'-dilithiobiphenyl and used it for bond length comparison.

Single crystals of **1** suitable for X-ray analysis were grown in the mixed solvents of hexane/THF (1:1) at -20 ºC. Single crystals of **2** and **3** suitable for X-ray analysis were grown in the mixed solvents of hexane/Et_2O/THF (10:10:1) at -20 ºC. Data collections were performed on a XtaLAB PRO 007HF(Mo): Kappa single diffractometer. Using Olex2[2], the structures were solved with the shelxs-97[3,4] program and refined with the XL refinement package[5] using Least Squares minimization. Refinement was performed on F^2 anisotropically for all the non-hydrogen atoms by the full-matrix least-squares method. The hydrogen atoms were placed at the calculated positions and were included in the structure calculation without further refinement of the parameters. Crystallographic data have been deposited with the Cambridge Crystallographic Data Centre as supplementary publication nos. CCDC 2033720 (**1**), CCDC 1984387 (**2**), CCDC 1984388 (**3**). Copies of these data can be obtained free of charge from the Cambridge Crystallographic Data Centre via www.ccdc.cam.ac.uk/data_request/cif.

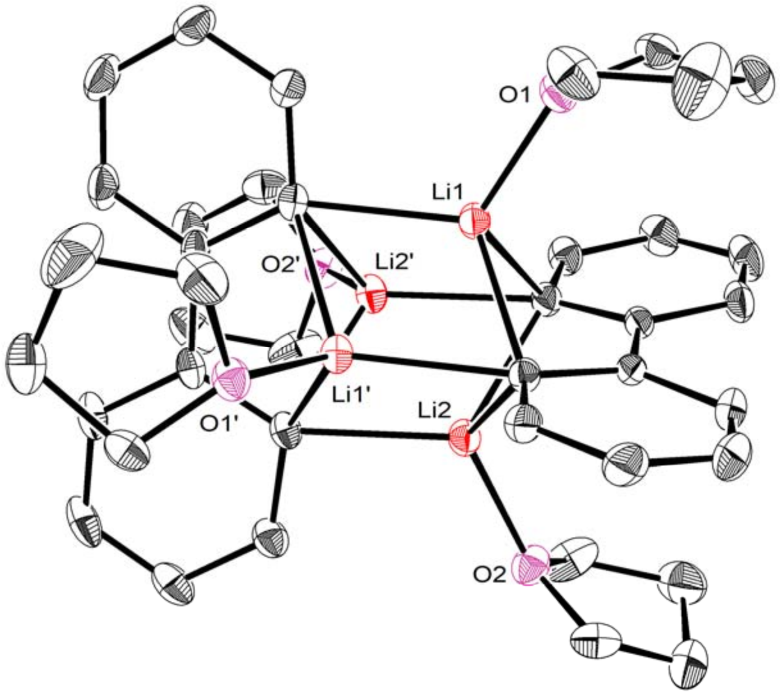

Supplementary Figure 1. ORTEP drawing of **1** with 30% thermal ellipsoids. Hydrogen atoms are omitted for clarity.

Supplementary Table 1. Crystal data and structure refinement for **1**.

Identification code	**1**
Empirical formula	$C_{40}H_{48}Li_4O_4$
Formula weight	620.54
Temperature/K	180.00(10)
Crystal system	monoclinic
Space group	I2/a
a/Å	22.5032(8)
b/Å	9.5181(3)
c/Å	17.0784(6)
α/°	90
β/°	105.617(4)
γ/°	90
Volume/Å^3	3522.9(2)
Z	4
ρ_{calc}g/cm^3	1.170
μ/mm^{-1}	0.071
F(000)	1328.0
Crystal size/mm^3	0.1 × 0.1 × 0.1
Radiation	MoKα (λ = 0.71073)
2Θ range for data collection/°	4.944 to 54.96
Index ranges	-27 ≤ h ≤ 29, -12 ≤ k ≤ 12, -22 ≤ l ≤ 22
Reflections collected	32738
Independent reflections	4032 [R_{int} = 0.0257, R_{sigma} = 0.0173]
Data/restraints/parameters	4032/0/217
Goodness-of-fit on F^2	1.033
Final R indexes [I>=2σ (I)]	R_1 = 0.0552, wR_2 = 0.1481
Final R indexes [all data]	R_1 = 0.0626, wR_2 = 0.1553
Largest diff. peak/hole / e Å^{-3}	0.47/-0.33

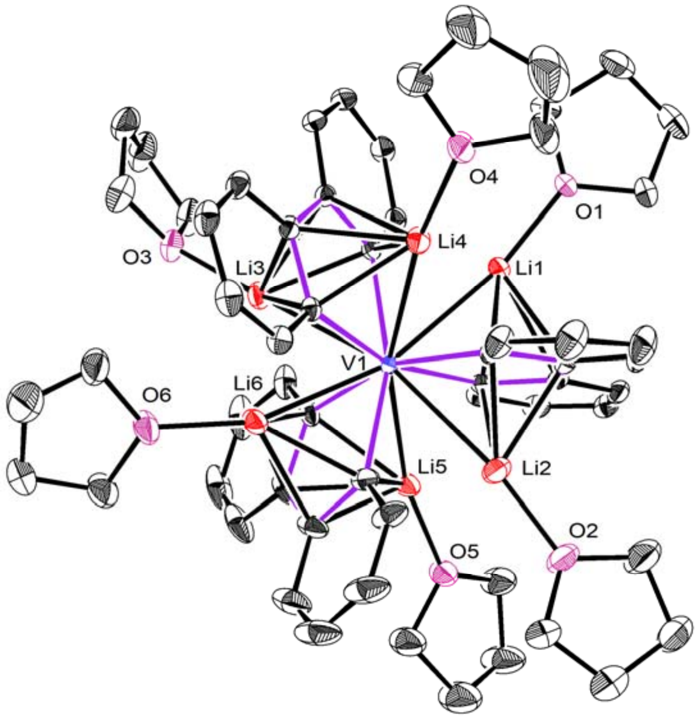

Supplementary Figure 2. ORTEP drawing of **2** with 30% thermal ellipsoids. Hydrogen atoms are omitted for clarity.

Supplementary Table 2. Crystal data and structure refinement for **2**.

Identification code	**2**
Empirical formula	$C_{60}H_{72}Li_6O_6V$
Formula weight	981.75
Temperature/K	180.00(10)
Crystal system	orthorhombic
Space group	Pbca
a/Å	16.8968(3)
b/Å	16.9241(3)
c/Å	36.5043(6)
α/°	90
β/°	90
γ/°	90
Volume/Å^3	10438.9(3)
Z	8
ρ_{calc}g/cm^3	1.249
μ/mm^{-1}	0.241
F(000)	4168.0
Crystal size/mm^3	0.2 × 0.2 × 0.1
Radiation	MoKα (λ = 0.71073)
2Θ range for data collection/°	4.776 to 54.968
Index ranges	$-21 \le h \le 21$, $-21 \le k \le 21$, $-47 \le l \le 47$
Reflections collected	203048
Independent reflections	11923 [R_{int} = 0.0654, R_{sigma} = 0.0236]
Data/restraints/parameters	11923/198/658
Goodness-of-fit on F^2	1.112
Final R indexes [I>=2σ (I)]	R_1 = 0.0775, wR_2 = 0.1875
Final R indexes [all data]	R_1 = 0.0906, wR_2 = 0.1966
Largest diff. peak/hole / e Å^{-3}	0.84/-0.68

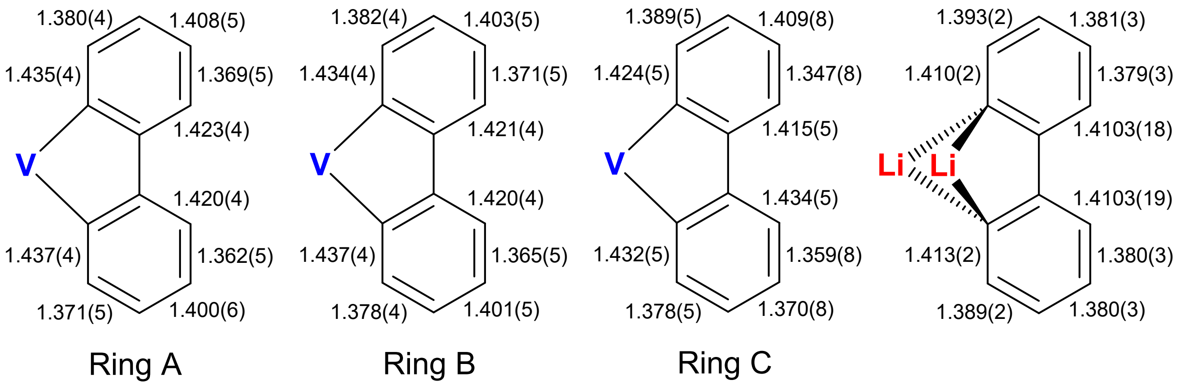

Supplementary Figure 3. C–C bond lengths (Å) of the ligands in complexes **2** and **1**.

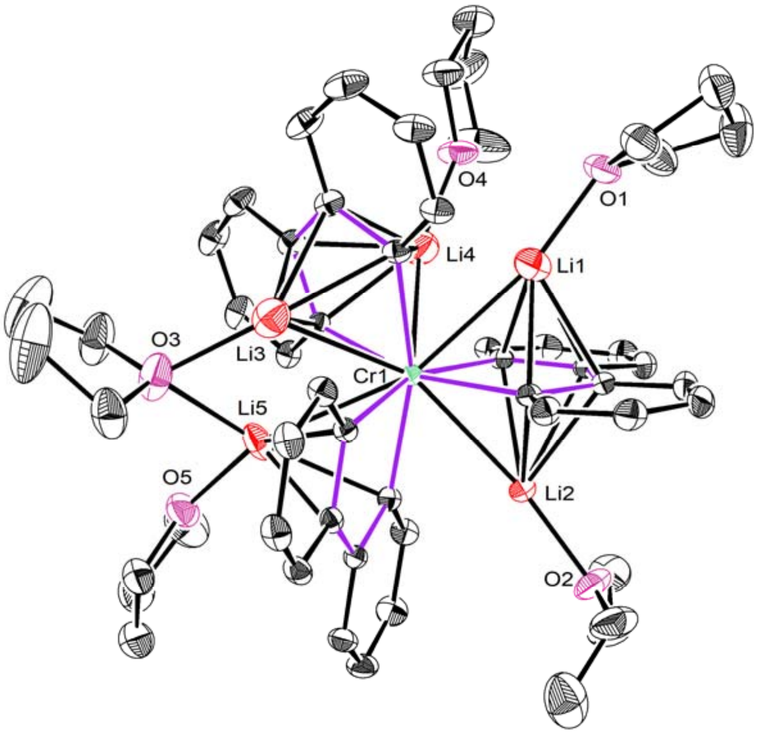

Supplementary Figure 4. ORTEP drawing of **3** with 30% thermal ellipsoids. Hydrogen atoms are omitted for clarity.

Supplementary Table 3. Crystal data and structure refinement for **3**.

Identification code	**3**
Empirical formula	$C_{119}H_{139}Cr_2Li_{10}O_{10}$
Formula weight	1902.69
Temperature/K	180.00(10)
Crystal system	monoclinic
Space group	$P2_1/c$
a/Å	10.0016(3)
b/Å	22.2190(6)
c/Å	23.5150(6)
α/°	90
β/°	100.696(3)
γ/°	90
Volume/Å^3	5134.8(3)
Z	2
ρ_{calc}g/cm^3	1.231
μ/mm^{-1}	0.271
F(000)	2022.0
Crystal size/mm^3	0.2 × 0.1 × 0.1
Radiation	MoKα (λ = 0.71073)
2Θ range for data collection/°	4.532 to 54.968
Index ranges	$-12 \leq h \leq 12$, $-28 \leq k \leq 27$, $-30 \leq l \leq 30$
Reflections collected	126378
Independent reflections	11726 [R_{int} = 0.0640, R_{sigma} = 0.0263]
Data/restraints/parameters	11726/0/673
Goodness-of-fit on F^2	1.093
Final R indexes [I>=2σ (I)]	R_1 = 0.0708, wR_2 = 0.1825
Final R indexes [all data]	R_1 = 0.0827, wR_2 = 0.1908
Largest diff. peak/hole / e Å^{-3}	1.25/-0.52

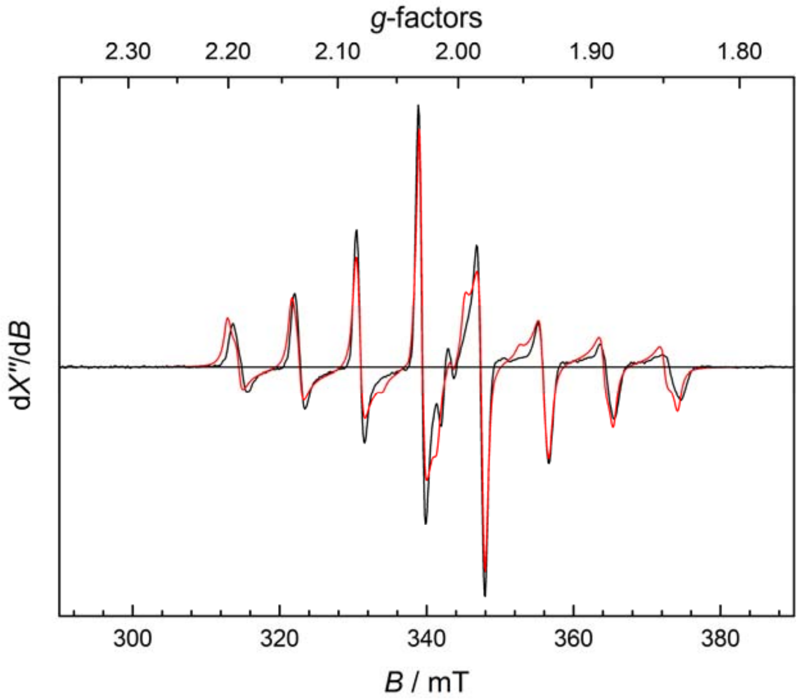

Supplementary Figure 5. X-band EPR spectra of complex **2** recorded at 30 K (black trace) and simulation (red trace).

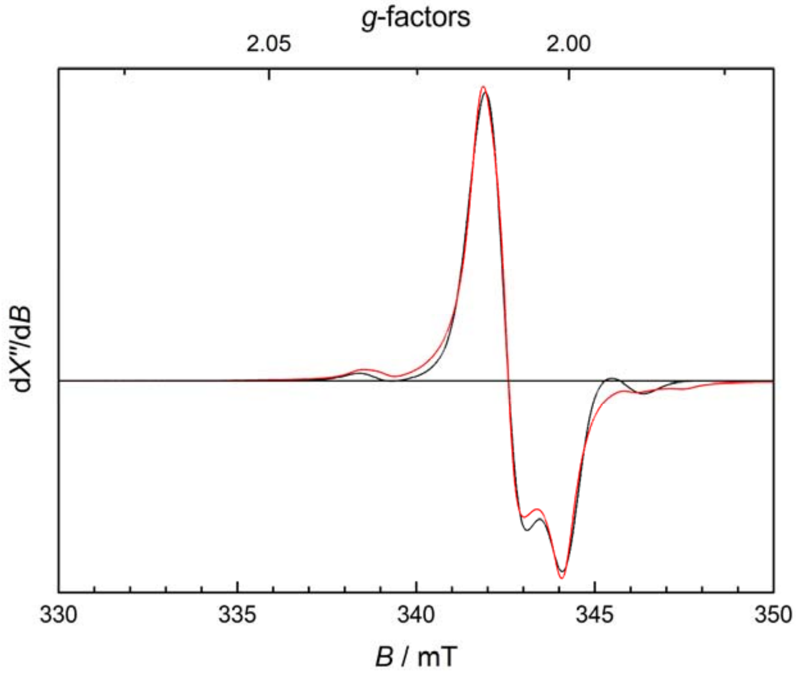

Supplementary Figure 6. X-band EPR spectra of complex **3** recorded at 40 K (black trace) and simulation (red trace).

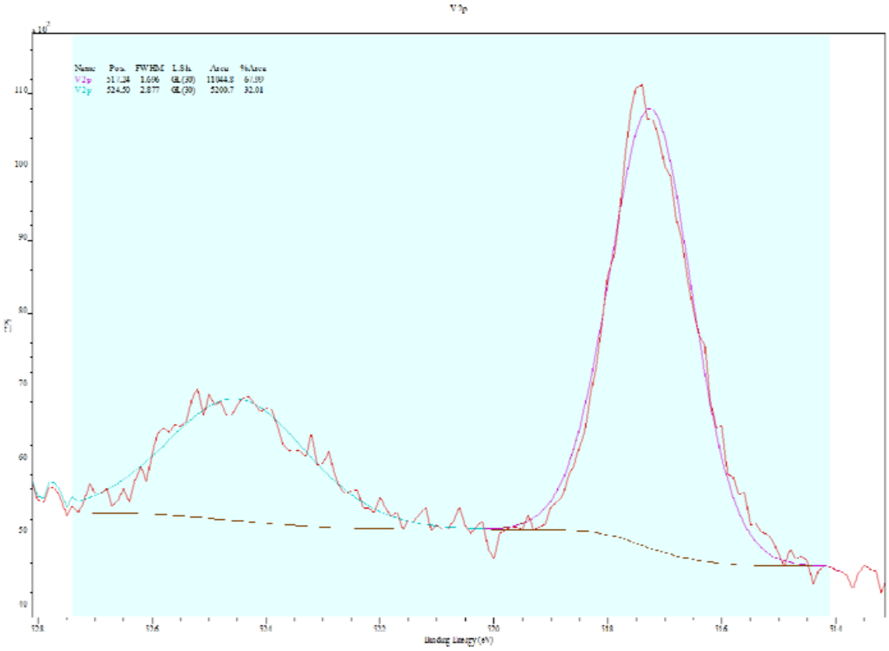

Supplementary Figure 7. XPS spectrum of V (2$p_{1/2}$ and 2$p_{3/2}$) in complex **2**.

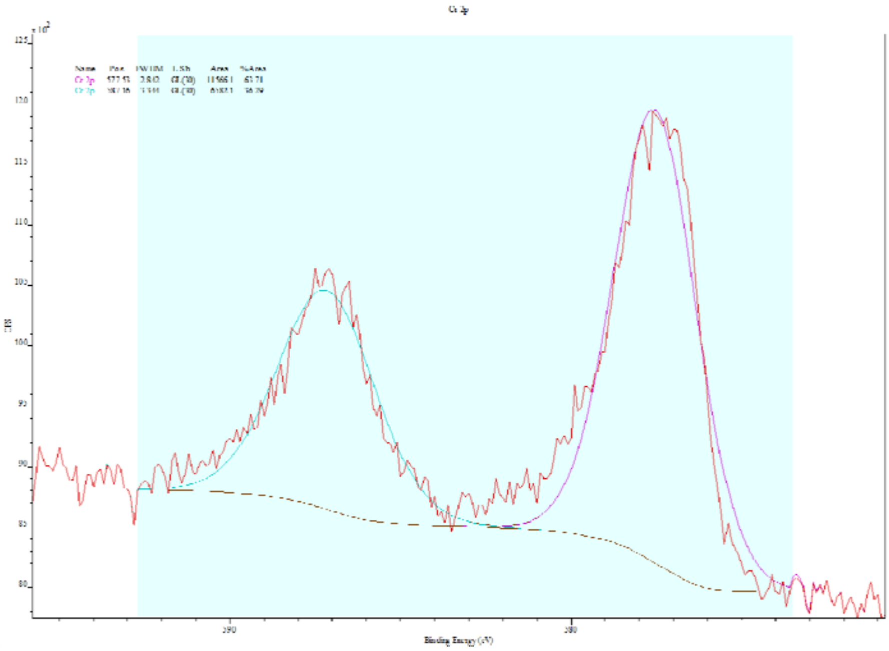

Supplementary Figure 8. XPS spectrum of Cr (2$p_{1/2}$ and 2$p_{3/2}$) in complex **3**.

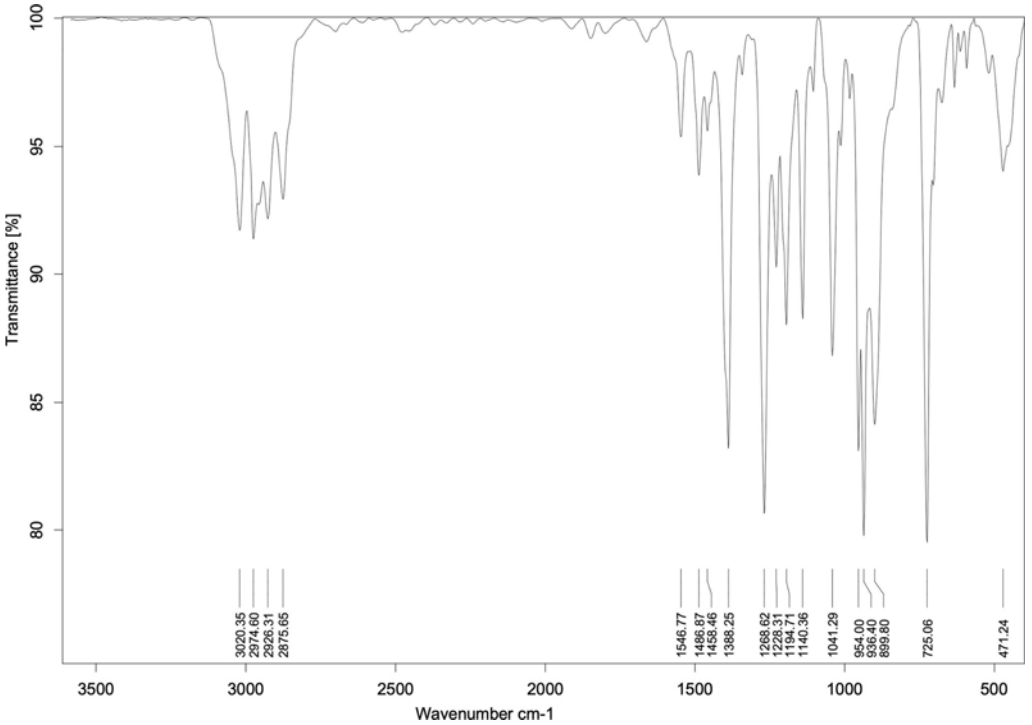

Supplementary Figure 9. IR spectrum of complex **2**.

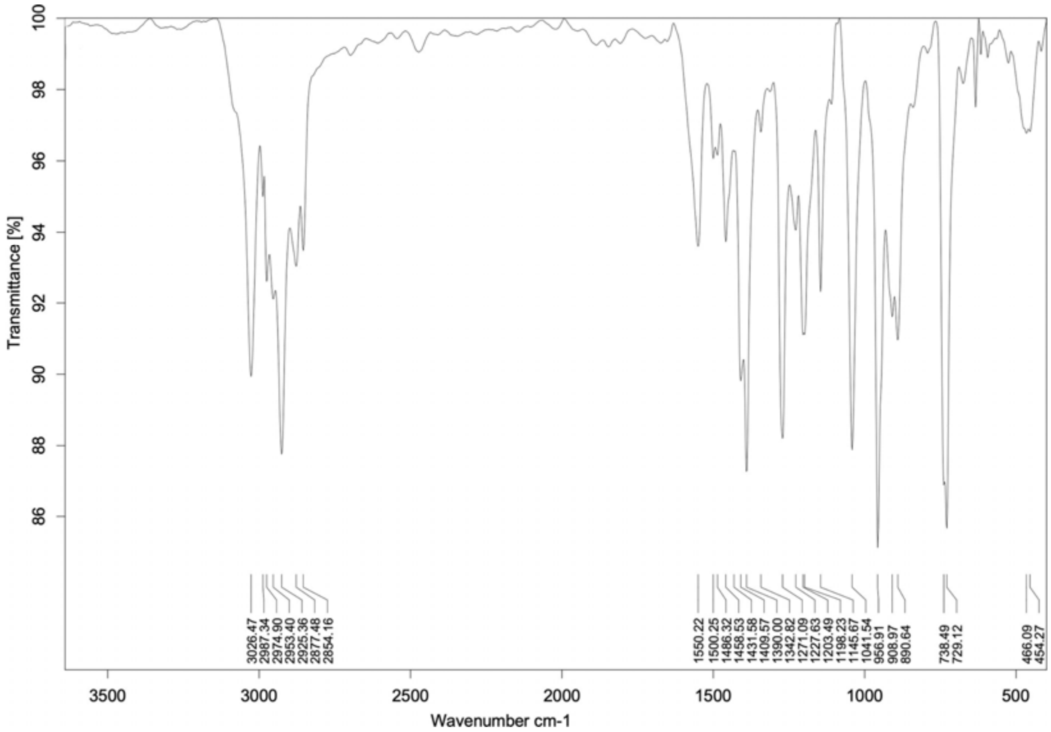

Supplementary Figure 10. IR spectrum of complex **3**.

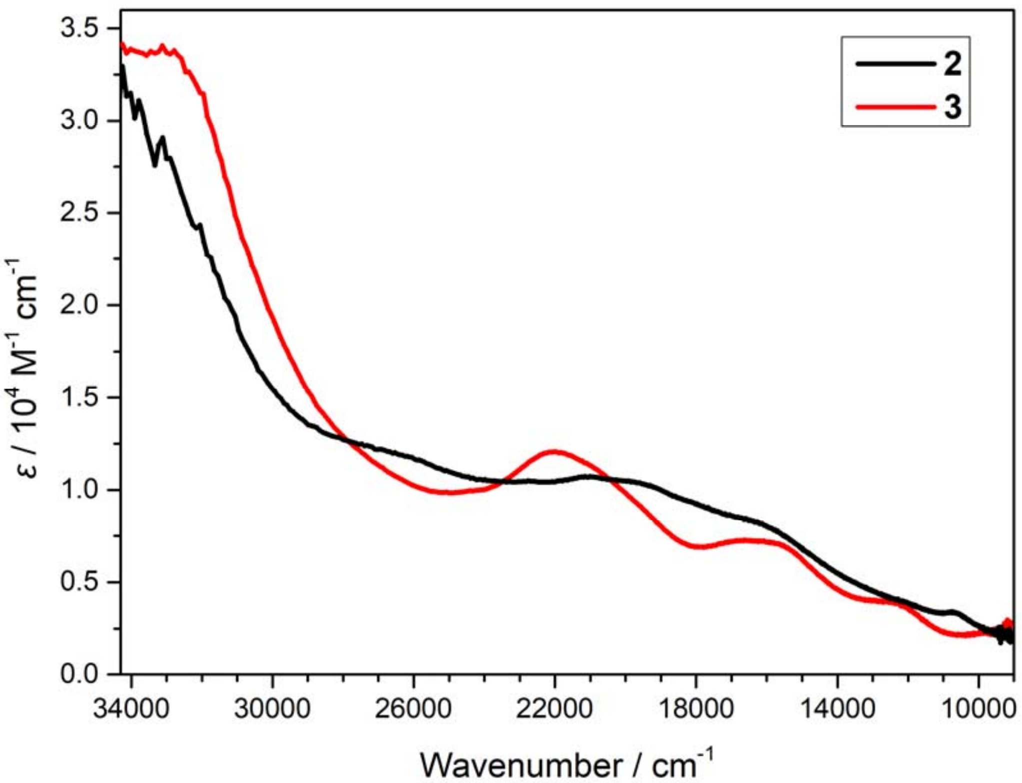

Supplementary Figure 11. UV-Vis spectra of complexes **2** and **3**.

Supplementary Table 4. Comparison of the bond lengths (Å) between the crystal structure and the theoretical structure of **2**.

		Crystal structure of **2**	Theoretical structure of **2**
Ring A	C_α–C_β	1.463(4), 1.462(4)	1.461, 1.461
	C_β–C_β	1.459(4)	1.459
	V–C_α	2.190(3), 2.189(3)	2.215, 2.204
Ring B	C_α–C_β	1.466(4), 1.470(4)	1.469, 1.466
	C_β–C_β	1.452 (4)	1.451
	V–C_α	2.188(3), 2.196(3)	2.204, 2.202
Ring C	C_α–C_β	1.474(5), 1.451(5)	1.464, 1.466
	C_β–C_β	1.459(6)	1.454
	V–C_α	2.195(3), 2.194(3)	2.208, 2.199

Supplementary Table 5. Comparison of the bond lengths (Å) between the crystal structure and the theoretical structure of **3**.

		Crystal structure of **3**	Theoretical structure of **3**
Ring A	C_α–C_β	1.440(4), 1.446(4)	1.453, 1.451
	C_β–C_β	1.470(4)	1.461
	Cr–C_α	2.150(2), 2.149(2)	2.154, 2.154
Ring B	C_α–C_β	1.452(4), 1.448(4)	1.473, 1.459
	C_β–C_β	1.461 (4)	1.448
	Cr–C_α	2.133(2), 2.145(3)	2.168, 2.179
Ring C	C_α–C_β	1.433(3), 1.425(3)	1.440, 1.432
	C_β–C_β	1.476(4)	1.472
	Cr–C_α	2.159(3), 2.139(2)	2.134, 2.150

Supplementary Table 6. Calculated NICS values of **2**-**5** (ppm).

		$NICS(1)_{zz}$	NICS(0)	$NICS(0)_{zz}$
**2**	Ring A	-24.9	-4.6	-6.9
	Ring B	-26.0	-5.1	-9.5
	Ring C	-24.4	-4.6	-7.7
**3**	Ring A	-18.5	-2.5	-2.4
	Ring B	-19.5	-2.9	-3.9
	Ring C	-3.1	2.1	8.9
**4**	Ring A	-16.9	-2.0	-1.4
	Ring B	-16.2	-2.2	-0.9
	Ring C	-16.5	-2.1	-1.8
**5**	Ring A	-26.5	-4.3	-7.9
	Ring B	-26.6	-5.2	-8.4
	Ring C	-8.1	1.2	5.9

Supplementary Table 7. Selected bond lengths (Å) of the optimized structures of **4** and **5**.

		**4**	**5**
Ring A	C_α–C_β	1.452, 1.451	1.475, 1.471
	C_β–C_β	1.461	1.448
	M–C_α	2.154, 2.161	2.215, 2.204
Ring B	C_α–C_β	1.450, 1.449	1.486, 1.477
	C_β–C_β	1.464	1.442
	M–C_α	2.156, 2.167	2.229, 2.240
Ring C	C_α–C_β	1.452, 1.453	1.444, 1.439
	C_β–C_β	1.460	1.475
	M–C_α	2.159, 2.159	2.202, 2.209

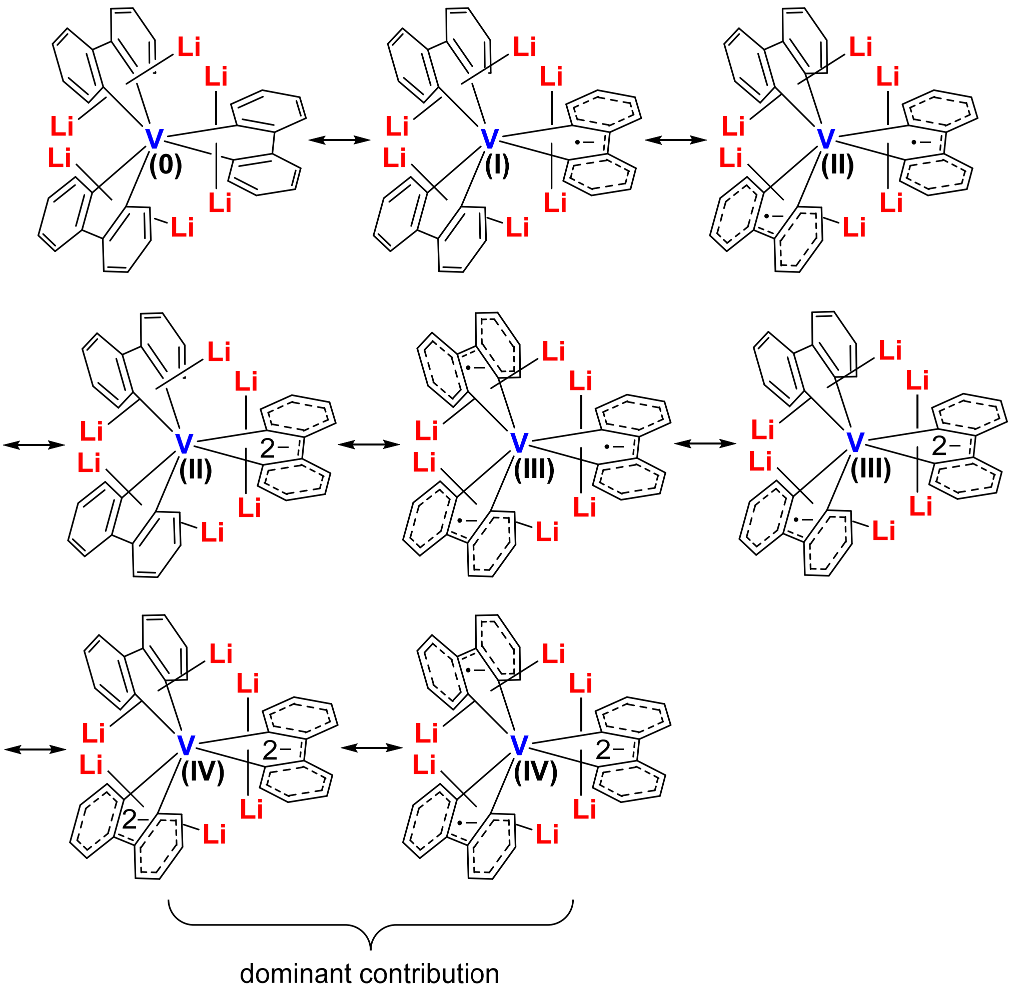

Supplementary Figure 12. Resonance structures of complex **2**.

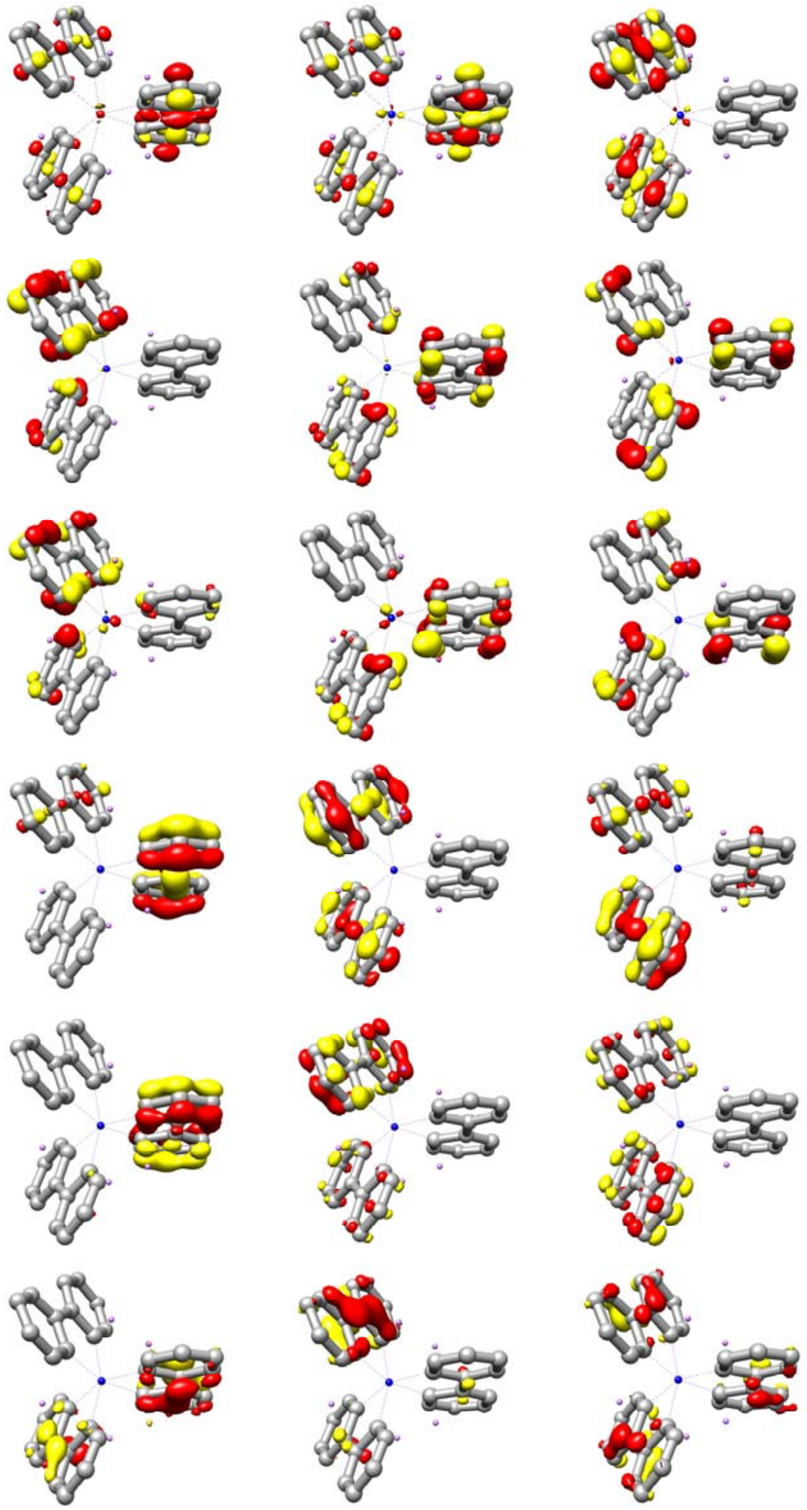

Supplementary Figure 13. The 18 pairs of π-Type MOs of complex **2**. As the spin-up and spin-down orbitals have spatial overlap **S** = 1, only the 18 spin-up MOs are shown here. The other four π-Type MOs are shown in Figure 3a.

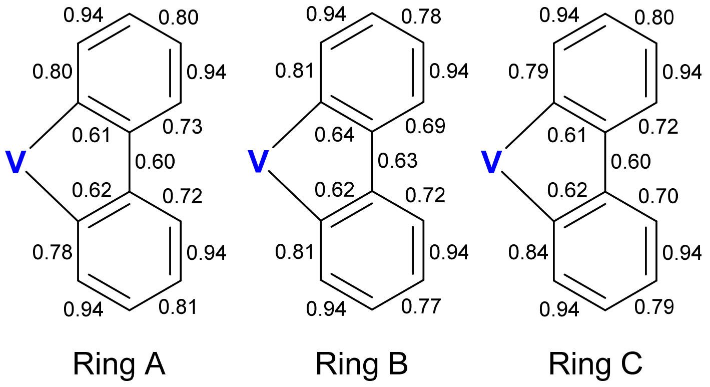

Supplementary Figure 14. ELF-π bifurcation values of the biphenyl ligands in complex **2**.

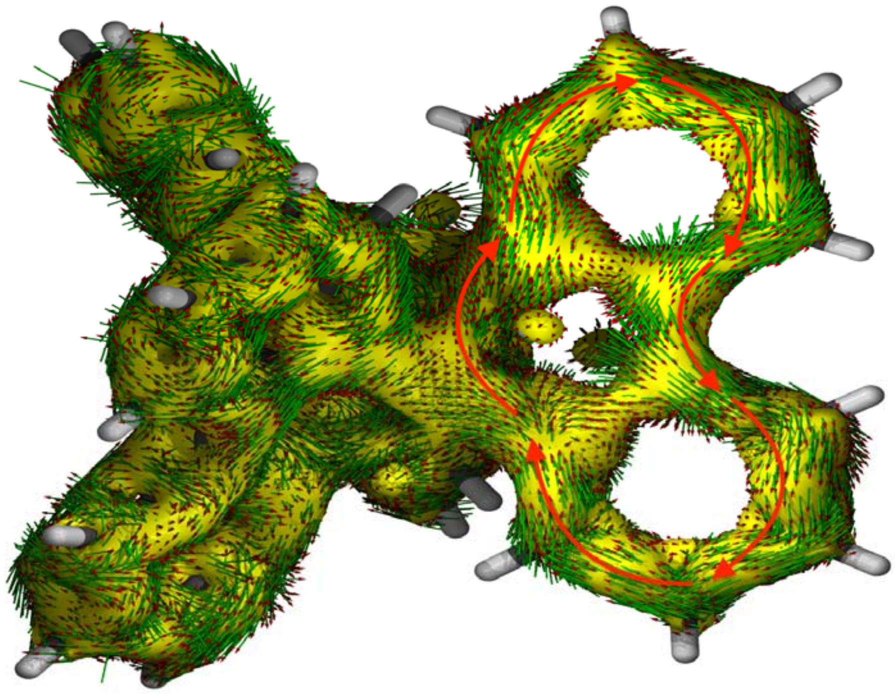

Supplementary Figure 15. Computed AICD plot of **2** from π contribution with an isosurface value of 0.03. The magnetic field vector is orthogonal with respect to the ring plane and points outside (clockwise currents are diatropic).

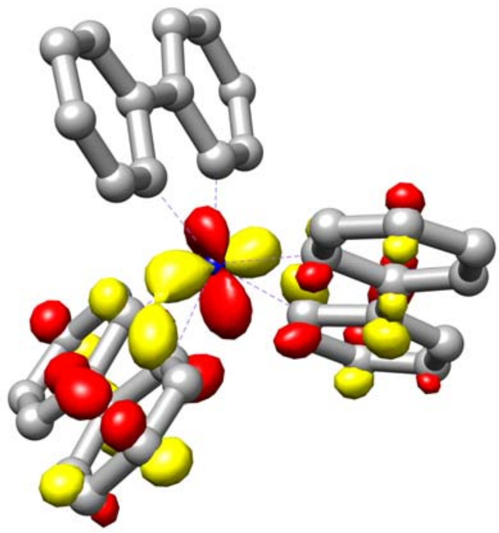

Supplementary Figure 16. The *d*-π* bonding orbital of the hypothetical hexaanion **2'**.

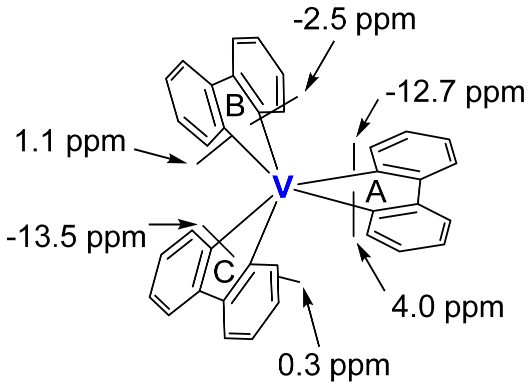

Supplementary Figure 17. The calculated NICS(1)$_{zz}$ values of the hypothetical hexaanion **2'**.

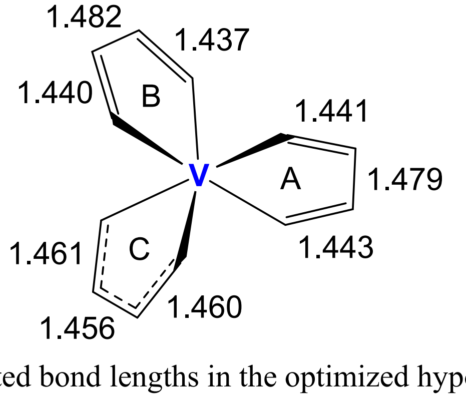

Supplementary Figure 18. Selected bond lengths in the optimized hypothetical hexaanion structure **2''**.

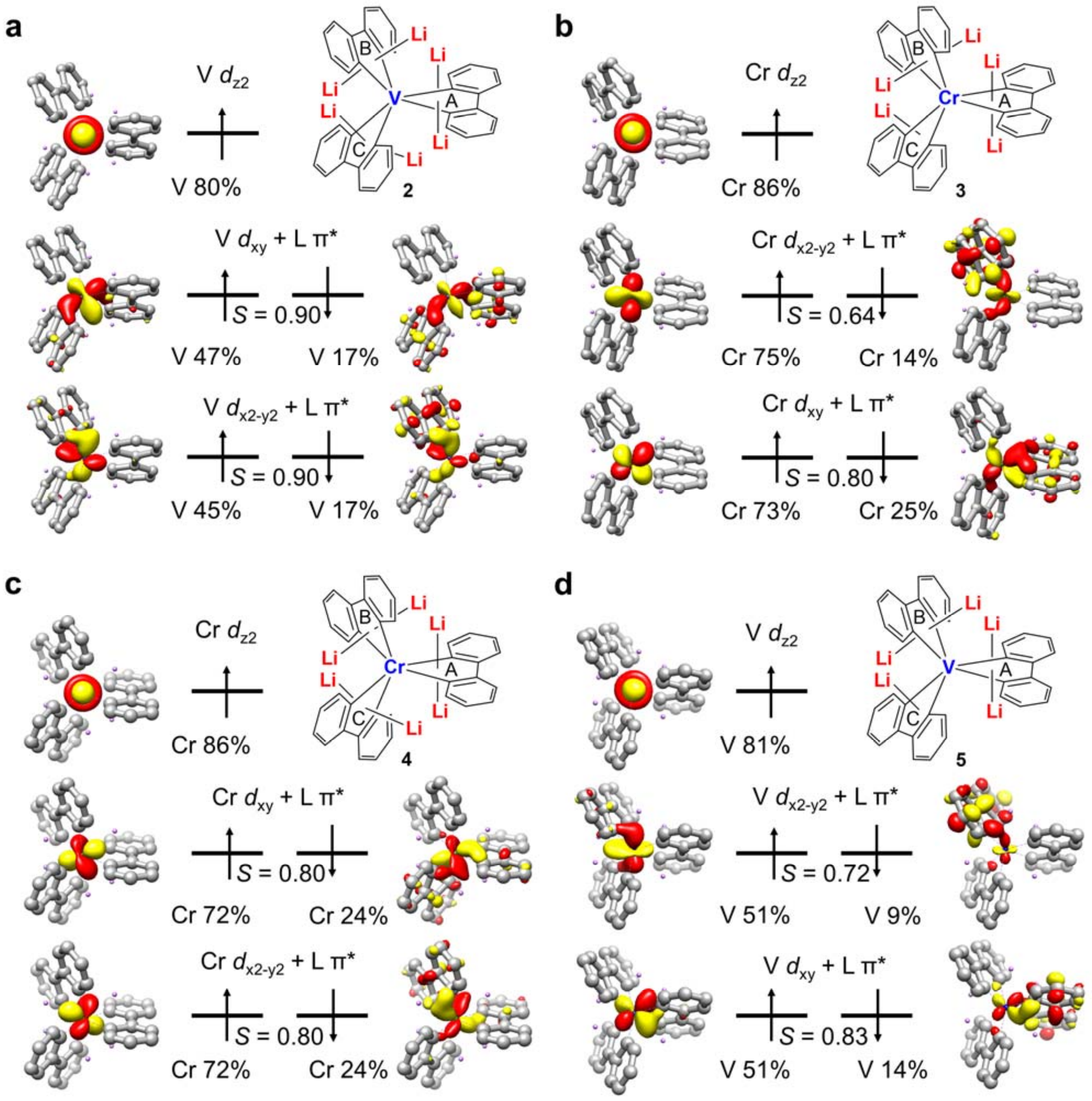

Supplementary Figure 19. Schematic MO diagrams of **2-5**. S is the spatial overlap between the two orbitals of a given spin coupled pair.

Supplementary References

1. Schubert, U., Neugebauer, W. & Schleyer, P. v. R. Symmetrical double lithium bridging in 2,2′-di(lithium-tmeda)biphenyl (tmeda = $MeNCH_2CH_2NMe_2$): experimental confirmation of theoretical predictions. *J. Chem. Soc., Chem. Commun.* 1184–1185 (1982).
2. Dolomanov, O. V., Bourhis, L. J., Gildea, R. J., Howard, J. A. K. & Puschmann, H. OLEX2: a complete structure solution, refinement and analysis program. *J. Appl. Cryst.* **42,** 339–341 (2009).
3. Sheldrick, G. M. A short history of SHELX. *Acta Crystallogr. A* **64,** 112–122 (2008).
4. Sheldrick, G. M. SHELXTL 5.10 for windows NT: *structure determination software programs*; bruker analytical X-ray systems, Inc.: Madison, WI, **1997**.
5. Sheldrick, G. M. Crystal structure refinement with SHELXL. *Acta Crystallogr. C.* **71,** 3–8 (2015).
