# Supplementary material for: A tris-spiro metalla-aromatic system featuring Craig-Möbius aromaticity
Source: Nat Commun. 2021 Feb 26;12:1319. doi: 10.1038/s41467-021-21648-9 (PMC7910433; doi:10.1038/s41467-021-21648-9)
Supplement: Supplementary file 2 — Description of Additional Supplementary Files [file 41467_2021_21648_MOESM2_ESM.pdf]

Description of Additional Supplementary Files

Supplementary Data 1: Cartesian coordinates of the optimized structures for DFT calculation
